# Susceptibility from the immunological perspective of COVID-19-associated pulmonary aspergillosis: A literature review

**DOI:** 10.1097/MD.0000000000042363

**Published:** 2025-05-09

**Authors:** Jiayin Wang, Xufeng Ji, Chun Yang, Jiancheng Xu

**Affiliations:** a Department of Laboratory, The First Hospital of Jilin University, Changchun, China; b Center of Infectious Diseases and Pathogen Biology, The First Hospital of Jilin University, Changchun, China.

**Keywords:** *Aspergillus*, CAPA, COVID-19-associated pulmonary aspergillosis, COVID-19 drug treatment, immuneresponse, SARS-CoV-2

## Abstract

The incidence rate of COVID-19-associated pulmonary aspergillosis (CAPA) is rising. However, the pathogenesis of CAPA remains unclear. Severe Acute Respiratory Syndrome Coronavirus 2 (SARS-CoV-2) infection disrupts pathways related to type I interferon and Toll-like receptors, key components in innate immunity, thereby elevating the incidence of CAPA. Additionally, SARS-CoV-2 infection results in T and B cell functional deficiencies or exhaustion within adaptive immunity, weakening the defense against invasive *Aspergillus*. Furthermore, SARS-CoV-2 infection enhances the replication of cytomegalovirus and alters the gut microbiota, factors that may aid in diagnosing CAPA. Immunosuppressive therapy in COVID-19 patients is also believed to heighten the risk of invasive aspergillosis. Therefore, this review, examines the immune response to SARS-CoV-2 infection combined with invasive aspergillosis, and explores the pathogenesis and susceptibility factors of CAPA. We propose that variations in an individual’s immune response significantly determine susceptibility to CAPA. The aim of this paper is to deepen clinical understanding of CAPA’s pathogenesis, thereby aiding in mitigating susceptibility risk and advancing novel treatment approaches.

## 1. Introduction

Infection with the Severe Acute Respiratory Syndrome Coronavirus 2 (SARS-CoV-2) through the respiratory tract leads to the development of Corona Virus Disease 2019 (COVID-19). While most COVID-19 patients are asymptomatic, some may develop acute respiratory distress syndrome and pneumonia.^[1,2]^ SARS-CoV-2 mutations contribute to increased immune suppression and a higher likelihood of prolonged hospitalization in patients. Consequently, this leads to increased vulnerability to fungal infections, potentially causing organ damage and even death.^[3,4]^ COVID-19-associated invasive fungal infections include COVID-19-associated pulmonary aspergillosis (CAPA), COVID-19-associated mucormycosis, and COVID-19-associated candidiasis.^[5]^ CAPA is the most prevalent complication among critically ill COVID-19 patients. This condition has an incidence rate of 5% to 35%, and its mortality rate is triple that of COVID-19 patients, especially in ICUs.^[6–10]^ It is crucial to understand the pathogenesis of SARS-CoV-2 virulence that leads to CAPA development.^[11]^ However, the pathophysiology of CAPA remains elusive, with COVID-19-associated systemic hyperinflammatory responses, lymphopenia, and immunological weaknesses contributing to an increased risk of *Aspergillus* infection. In light of the limited understanding of CAPA pathogenesis, this review synthesizes literature on SARS-CoV-2 and invasive *Aspergillus* coinfections, focusing on their interrelated immune responses. It outlines the indicators of immune changes in CAPA patients and the immune mechanisms by which COVID-19 therapeutic agents heighten CAPA risk.

## 2. CAPA is influenced by innate immunity

**Figure 1.**
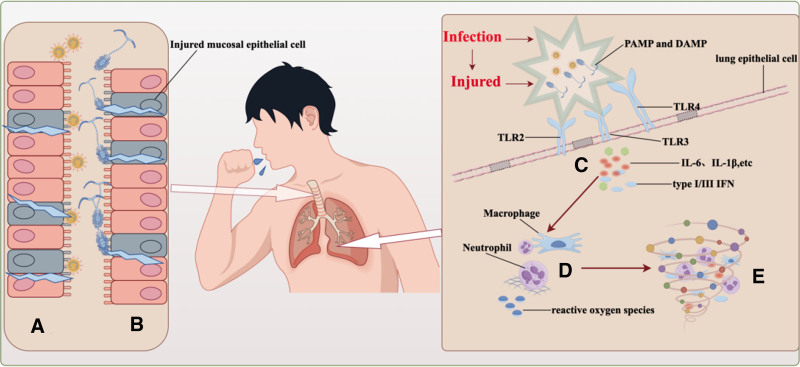
(A) After SARS-CoV-2 infection, respiratory epithelial cells are destroyed. (B) Invasive *Aspergillus* infection is facilitated by damaged respiratory epithelial cells. (C) SARS-CoV-2 inhibits type I and III interferons required for fungal immunity. (D) SARS-CoV-2 decreases antifungal immunity by inhibiting the activity of alveolar macrophages and neutrophils. (E) Patients with severe COVID-19 may have increased immune system activation, which can cause cytokine storms and weaken their immunological defenses against invasive *Aspergillus* infection. COVID-19 = Corona Virus Disease 2019, SARS-CoV-2 = Severe Acute Respiratory Syndrome Coronavirus 2.

Figure 1 depicts a simplified example of innate immunity’s role in the pathogenesis of CAPA. After SARS-CoV-2 infection, the virus binds to airway epithelial cells via ACE2, replicating and multiplying, and infecting ciliated cells. This leads to the dedifferentiation of multiciliated cells and loss of mucosal clearance function.^[12,13]^ The preservation of the innate immunological barrier in respiratory epithelial cells is crucial to protect against *Aspergillus* infection. Epithelial cells form a complete physical barrier against *Aspergillus*, removing conidia through phagocytosis and mucus cilia. In the aftermath of SARS-CoV-2 infection, *Aspergillus* mycelium infiltrates the mucosa, destroying epithelial cells. This breakdown is a significant factor in the progression from COVID-19 to CAPA in patients.^[14–17]^

After the virus destroys mucosal epithelial cells, the innate immune system detects PAMPs via PRRs on immune cells. This activates PRRs and related anti-inflammatory pathways, leading to cytokine and IFN production.^[18]^ For instance, SARS-CoV-2 inhibits type I IFN expression by disrupting the downstream cytoplasmic RNA sensor signaling pathway. Additionally, the structural characteristics of SARS-CoV-2 contribute to the inhibition of type I IFN production.^[19–21]^ Type I IFN is critical for resistance to *Aspergillus* infection, and the absence of type III IFN suppresses neutrophil response against *Aspergillus*.^[22,23]^ The severity of COVID-19 is closely linked to the suppression of the type I IFN-mediated antiviral response, potentially contributing to the prevalence of CAPA in severe cases.^[24]^ TLR2, TLR3, and TLR4, members of the PRR family of Toll-like receptors, are associated with SARS-CoV-2 and *Aspergillus* coinfection. SARS-CoV-2 infection triggers the expression of TLR2, TLR3, and TLR4. TLR2 and TLR4 mainly recognize viral envelope proteins, while TLR3 identifies double-stranded RNA during viral replication.^[25,26]^ However, TLR2 and TLR4 are involved in the immune response to *Aspergillus* infection. TLR2 is part of the body’s rapid response to *Aspergillus* invasion. TLR4 mediates monocyte recognition of *Aspergillus* filaments, and *Aspergillus* cell wall components diminish TLR4-mediated immune responses.^[27–30]^ TLR3 primarily recognizes *Aspergillus fumigatus* RNA, and its knockout heightens *Aspergillus* infection risk in mice. The immune recognition pathway activated post-SARS-CoV-2 infection might interfere with *Aspergillus* invasion recognition, potentially creating a vicious cycle of immune response between the two.

Additionally, cells infected with SARS-CoV-2 produce DAMPs, detected by PRRs and activating alveolar macrophages, neutrophils, and other immune cells. SARS-CoV-2 infection impairs the function of these cells, leading to irreversible lung damage.^[31–33]^ Individuals with severe COVID-19, exhibiting high immune response and pro-inflammatory cytokine production, may trigger cytokine storms, leading to immunological insufficiency or paralysis.^[34,35]^ However, the body’s defense against *Aspergillus* involves alveolar macrophages, neutrophils, and other immune cells. Neutrophils create extracellular traps and generate reactive oxygen species, while alveolar macrophages eliminate *Aspergillus* conidia through phagocytosis and release pro-inflammatory cytokines.^[36,37]^ Consequently, cytokine storms heighten the risk of CAPA susceptibility.

## 3. CAPA is influenced by adaptive immunity

Adaptive immunity is critical for SARS-CoV-2 viral clearance and *Aspergillus* invasion resistance. T and B lymphocytes, responsible for adaptive immunity, initiate a specific response upon stimulation by innate immune-activating cytokines, leading to lymphocyte proliferation and activation.

**Figure 2.**
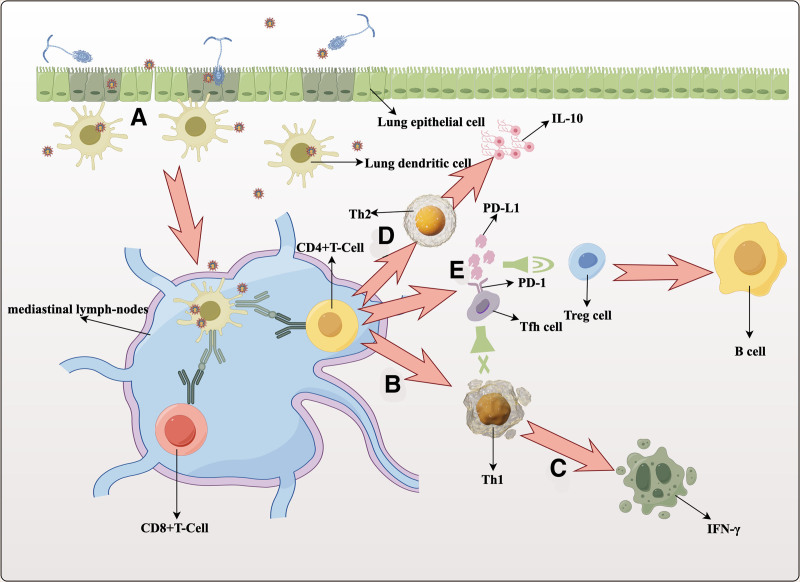
(A) During SARS-CoV-2 infection, lung epithelial cells are continuously destroyed, and *Aspergillus* invasion with spores releases molecules that may promote membrane permeability and tissue damage, which again destroys lung epithelial cells. (B) The SARS-CoV-2 virus inhibits Th1 via CD4+ T cells. (C) Th1 secretes cytokine IFN-γ. (D) Th2 secretion of IL-10 is increased in COVID-19 patients, and increased IL-10 increases the risk of invasive *Aspergillus* infections. (E) SARS-CoV-2 infection increased the expression of PD-1, and invasive *Aspergillus* infection increased the expression of PD-L1. The interaction between PD-1 and PD-L1 promoted Treg cell response and inhibited Th1 response. SARS-CoV-2 = Severe Acute Respiratory Syndrome Coronavirus 2.

Figure 2 depicts a simplified example of adaptive immunity’s role in the pathogenesis of CAPA. SARS-CoV-2 infects lung dendritic cells and spreads to mediastinal lymph nodes, where antigen-presenting cells facilitate its attachment to T cells, stimulating their differentiation into CD4+ and CD8+ T cells. SARS-CoV-2 infection suppresses type I helper T-lymphocytes. The differentiation of CD4+ T-cells from these lymphocytes leads to IFN-γ production, crucial for resistance to *Aspergillus* invasion. The use of anti-IFN-γ antibodies in invasive pulmonary aspergillosis patients has been shown to exacerbate the disease.^[38]^ Furthermore, IL-10, produced by type II helper T-lymphocytes, warrants special attention. Research indicates that COVID-19 patients show significantly higher serum IL-10 levels in the disease’s early stages, preceding the increase of other cytokines. IL-10 serves as an anti-inflammatory cytokine and an indicator of immunosuppression.^[39–41]^ IL-10 plays a role in the host’s defense against *Aspergillus* invasion during disease progression. In patients with confirmed invasive *Aspergillus* infections, a high IFN-γ/IL-10 ratio suggests effective therapy, indicating a connection between elevated IL-10 and the progression of these infections.^[42]^

T follicular helper cells (Tfh) from CD4+ T cells mediate B cell differentiation into plasma cells by enhancing their adhesion to B cells.^[43]^ Characteristic surface receptors of Tfh cells include programmed cell death protein 1 (PD-1). SARS-CoV-2 infection elevates PD-1 expression, indicative of T-cell exhaustion.^[44–46]^ α-(1,3) glucan in *Aspergillus* cell walls triggers heightened PD-L1 expression. The PD-1 and PD-L1 interaction promotes regulatory T cell responses, while suppressing type I helper T lymphocyte responses.^[47,48]^ Regulatory T cells mitigate inflammation and tissue damage during infection, suppress protective immune responses, and enhance persistence of conditions.^[49]^ The immunological interactions between SARS-CoV-2 and invasive *Aspergillus* coinfection, as well as T-cell impairment caused by SARS-CoV-2, escalate the risk of CAPA.

## 4. Immunity indicators influence CAPA

### 4.1. Cytomegalovirus

Cytomegalovirus (CMV) infections commonly occur in immunocompromised individuals, including organ transplant recipients and AIDS patients.^[50]^ CMV infection suppresses the immune response and elevates the risk of opportunistic fungal infections in organ transplant patients.^[51]^ Studies have demonstrated a direct correlation between CMV load in the blood and the risk of invasive *Aspergillus* infection.^[52]^ The amplification of CMV replication may result from lung injury and immune deficiency induced by SARS-CoV-2 infection.^[53]^ Consequently, CMV infection may be a contributing risk factor for CAPA.

Calderón-Parra J et al found that in CAPA patients, CMV exhibits higher replication frequency and viral load, often preceding the diagnosis of CAPA.^[54]^ The concurrent infections of CMV and SARS-CoV-2 may synergistically increase susceptibility to and exacerbate the progression of CAPA. Diagnosing CAPA remains challenging, necessitating additional research into the correlation between CMV markers and CAPA infection. The escalation of serum CMV levels alongside replication in COVID-19 patients indicates an increased risk of CAPA infection, underscoring the need for early prevention and management to mitigate CAPA development.

### 4.2. Gut microbiota

The gut microbiota plays a crucial role in the immunological defense of gut lymphocytes and interacts with host immune cells, influencing the innate and adaptive immune systems.^[55–57]^ SARS-CoV-2 impacts gut microbiota through various pathways, such as altering intestinal permeability, nutrient transport, and inducing local and systemic inflammation. For instance, SARS-CoV-2 influences gut microbiota by downregulating ACE2 receptors on intestinal epithelial cells and via the bidirectional gut–lung axis connecting gut microbiota and the lungs.^[58–61]^ However, a reciprocal regulatory relationship is observed between gut bacterial metabolites and *Aspergillus* immunity.^[62]^

Animal studies reveal that gut microbiota modulates the adaptive immune response in the lungs to *Aspergillus fumigatus* by regulating CD4+ T cells.^[63,64]^ Maurer HC et al observed that CAPA patients exhibit a trend towards reduced gut microbial diversity, while *Staphylococcus epidermidis* abundance increases in early-stage patients.^[65]^ This suggests that alterations in gut microbiota accompany severe CAPA and potentially impact the host’s immune response. Despite the clinical study not eliminating potential interference from antifungal drugs and limitations due to a small sample size, it offers valuable insights for CAPA diagnosis and treatment.

## 5. Therapeutic drugs influence CAPA

### 5.1. Corticosteroids

Patients with severe COVID-19 display significant inflammatory responses, and corticosteroid therapy has been shown to reduce overall mortality by 10% to 30% in these patients.^[66]^ The WHO recommends corticosteroid therapy for COVID-19 patients needing respiratory support.^[67,68]^ Nevertheless, a growing body of research indicates that COVID-19 patients treated with corticosteroids face a higher risk of CAPA. Erami M et al found that COVID-19 patients frequently treated with corticosteroids were more likely to develop CAPA, based on a comparison of baseline characteristics between CAPA and non-CAPA patients. Leistner et al demonstrated that corticosteroids, particularly dexamethasone, are a risk factor for CAPA, tripling the risk.^[69–72]^

Corticosteroids reduce macrophage phagocytosis by promoting neutrophil apoptosis and dampening the immune responses of T and B cells, including CD4 and Th1 cells. Thus, while corticosteroids reduce excessive inflammation, they concurrently suppress immune responses and pathogen clearance.^[73]^ This immunosuppressive action impairs the host’s antifungal immune response by inhibiting T cell activation, a risk factor for invasive *Aspergillus* infection. Additionally, corticosteroids enhance the germination of extracellular conidia on bronchial epithelial cells by suppressing IFN production and function, inducing cytokine signaling, downregulating IFN-λ gene expression and translation, and inhibiting the PI3K signaling pathway. Prolonged and continuous use of high-dose corticosteroids leads to upregulation of ACE2 receptors in COVID-19 patients. Given that ACE2 receptors are pivotal in binding or enhancing the germination of extracellular spores, corticosteroids significantly elevate the risk of *Aspergillus* infection in COVID-19 patients.^[74–76]^

### 5.2. Anti-IL-6 drugs

Monoclonal antibody drugs, such as tocilizumab, target interleukin-6 (IL-6) receptors, primarily inhibiting the function of IL-6. Under healthy conditions, IL-6 levels are lower, playing a key role in immunoregulation. Following SARS-CoV-2 infection, an overreactive immune response releases substantial IL-6, potentially inducing a cytokine storm. Tocilizumab, by inhibiting IL-6, enhances the immune response in COVID-19 patients. Such drugs offer a potential treatment strategy for severe COVID-19 cases.^[77,78]^

Gupta S et al reported that COVID-19 patients treated with tocilizumab had a reduced risk of hospitalization.^[79]^ However, anti-IL-6 drugs may diminish the Th17 immune response and the efficacy of phagocytic cells, leading to impaired recruitment of AMP and neutrophils vital for antifungal defense.^[80,81]^ Theoretically, the use of anti-IL-6 drugs heightens the susceptibility of COVID-19 patients to *Aspergillus* infection. In fact, a retrospective study revealed that tocilizumab-treated COVID-19 patients had a higher tendency for fungal infections and increased mortality.^[82]^ Other studies have indicated that tocilizumab treatment in COVID-19 patients elevates the risk of CAPA, with 1 prospective study noting a 100% mortality rate post-*Aspergillus* infection in such patients.^[83–86]^ While some studies suggest an increased risk of CAPA development in COVID-19 patients treated with anti-IL-6 drugs, more research is required to understand their immunological impact on CAPA. This will enhance the clinical management of COVID-19 and invasive *Aspergillus* infections through more judicious drug use.

## 6. Future perspectives

With the easing of COVID-19 lockdown measures worldwide, there is a rapid increase in the number of COVID-19 cases. Urgent measures are imperative for the timely diagnosis and effective prevention of CAPA, necessitating a thorough understanding of its underlying immune mechanisms. Presently, research on the immune mechanisms of CAPA is limited. This review summarizes the potential interactions and mechanisms between SARS-CoV-2 and invasive *Aspergillus*. Certain pathways in both innate and adaptive immunity may be crucial in CAPA development. Variations in individual immune function result in diverse immune responses to SARS-CoV-2. A strong correlation exists between the immune response to SARS-CoV-2 and susceptibility to CAPA. Diagnosing CAPA continues to be challenging, necessitating differentiation between *Aspergillus* colonization and invasive disease. Presently, heightened CMV replication and diminished gut microbiota are associated with CAPA.

Further research is expected to clarify their role in CAPA diagnosis. Moreover, therapeutic drugs used in COVID-19, including corticosteroids and anti-IL-6 medications, increase the risk of invasive *Aspergillus* infection. This highlights the criticality of selecting appropriate therapeutic drugs for COVID-19 patients. It is essential to note the potential adverse effects of current drugs on the immune pathways, which may heighten susceptibility to invasive *Aspergillus*. Additional prospective studies are essential to provide dependable data for more informed drug use in the future.

## Author contributions

**Conceptualization:** Jiayin Wang, Xufeng Ji, Jiancheng Xu.

**Investigation:** Jiayin Wang, Xufeng Ji, Jiancheng Xu.

**Funding acquisition:** Jiancheng Xu.

**Methodology:** Jiancheng Xu.

**Supervision:** Chun Yang.

**Writing – original draft:** Jiayin Wang, Xufeng Ji.

**Writing – review & editing:** Chun Yang, Jiancheng Xu.
